# Engineered PAM-SPION Nanoclusters for Enhanced Cancer Therapy: Integrating Magnetic Targeting with pH-Responsive Drug Release

**DOI:** 10.3390/molecules30132785

**Published:** 2025-06-28

**Authors:** Dimitra Tzavara, Konstantina Papadia, Argiris Kolokithas-Ntoukas, Sophia G. Antimisiaris, Athanasios Skouras

**Affiliations:** 1 Laboratory of Pharmaceutical Technology, Department of Pharmacy, School of Health Sciences, University of Patras, 26504 Rio Patras, Greece; tzavaradim@gmail.com (D.T.); kon.papadia@gmail.com (K.P.); argiris9495@gmail.com (A.K.-N.); santimis@upatras.gr (S.G.A.); 2 Regional Centre of Advanced Technologies and Materials, Czech Advanced Technology and Research Institute (CATRIN), Palacký University Olomouc, 779 00 Olomouc, Czech Republic; 3 Institute of Chemical Engineering Sciences, Foundation for Research and Technology Hellas (FORTH/ICES), 26504 Rio Patras, Greece

**Keywords:** superparamagnetic iron oxide nanoparticles, magnetic targeting, drug delivery, pH-responsive, cancer therapy, alternating magnetic field, hyperthermia, theranostic

## Abstract

Background: Nanomedicine approaches for cancer therapy face significant challenges, including a poor tumor accumulation, limited therapeutic efficacy, and systemic toxicity. We hypothesized that controlling the clustering of poly(acrylic acid-co-maleic acid) (PAM)-coated superparamagnetic iron oxide nanoparticles (SPIONs) would enhance their magnetic properties for improved targeting, while enabling a pH-responsive drug release in tumor microenvironments. Methods: PAM-stabilized SPION clusters were synthesized via arrested precipitation, characterized for physicochemical and magnetic properties, and evaluated for doxorubicin loading and pH-dependent release. A dual targeting approach combining antibody conjugation with magnetic guidance was assessed in cellular models, including a novel alternating magnetic field (AMF) pre-treatment protocol. Results: PAM-SPION clusters demonstrated controlled size distributions (60–100 nm), excellent colloidal stability, and enhanced magnetic properties, particularly for larger crystallites (13 nm). The formulations exhibited a pH-responsive drug release (8.5% at pH 7.4 vs. 14.3% at pH 6.5) and a significant enhancement of AMF-triggered release (17.5%). The dual targeting approach achieved an 8-fold increased cellular uptake compared to non-targeted formulations. Most notably, the novel AMF pre-treatment protocol demonstrated an 87% improved therapeutic efficacy compared to conventional post-treatment applications. Conclusions: The integration of targeting antibodies, magnetic guidance, and a pH-responsive PAM coating creates a versatile theranostic platform with significantly enhanced drug delivery capabilities. The unexpected synergistic effect of the AMF pre-treatment represents a promising new approach for improving the therapeutic efficacy of nanoparticle-based cancer treatments.

## 1. Introduction

Cancer remains one of the leading causes of mortality worldwide, with conventional chemotherapeutic approaches limited by poor tumor selectivity, dose-limiting toxicities, and the development of drug resistance [1,2]. Nanomedicine approaches have shown promise in addressing these challenges, offering the potential for targeted delivery, controlled release, and multi-modal therapeutic strategies [3,4]. Among various nanoplatforms, superparamagnetic iron oxide nanoparticles (SPIONs) have emerged as particularly versatile candidates for biomedical applications due to their unique magnetic properties, biocompatibility, and potential for both diagnostic imaging and therapeutic interventions [5,6].

SPIONs’ ability to respond to external magnetic fields enables both diagnostic imaging and therapeutic applications through magnetic targeting and hyperthermia. When exposed to external magnetic fields, SPIONs can be directed to specific tissues, while under alternating magnetic fields, they generate heat that can selectively damage cancer cells or trigger a controlled drug release [7]. This dual functionality positions SPIONs as ideal candidates for theranostic applications, combining diagnostic capabilities with therapeutic interventions in a single platform.

The surface coating of SPIONs is critical for ensuring colloidal stability, biocompatibility, and functionality in biological environments. Uncoated SPIONs rapidly aggregate and are quickly cleared by the mononuclear phagocyte system, resulting in limited therapeutic efficacy [8,9]. Surface modification not only prevents aggregation but also provides reactive groups for conjugating targeting ligands and therapeutic agents, transforming simple nanoparticles into multifunctional platforms [10,11].

Despite significant progress, current SPION-based systems face several limitations. Developing truly multi-modal systems with integrated diagnostic, therapeutic, and targeting capabilities often results in compromised stability and unpredictable in vivo behavior [12,13]. Existing targeting strategies also present challenges: passive targeting via an enhanced permeability and retention effect shows limited clinical efficacy due to tumors’ heterogeneity [14], while active targeting using ligands faces challenges, including immunogenicity and poor tissue penetration [15]. Magnetic targeting offers a promising alternative but requires precise control over magnetic field parameters and particle properties to achieve effective delivery, particularly to deep tissues [16].

Similarly, magnetic hyperthermia applications are limited by the need for an optimal crystallite size to maximize heat generation capacity, as well as difficulties in precisely controlling the temperature distribution within tissues [17]. The therapeutic window between effective hyperthermia (>41 °C) and damaging healthy tissues is narrow, necessitating the careful optimization of both nanoparticle properties and field application parameters. Recent advances in understanding magnetic relaxation mechanisms have shown that nanoparticle size, crystallinity, and magnetic anisotropy significantly impact heating efficiency and MRI contrast properties [18,19].

Poly(acrylic acid-co-maleic acid) (PAM) represents a promising coating material to address these challenges. Recent research has demonstrated its effectiveness as a coating for various types of metal oxide nanoparticles [20]. The copolymer structure of PAM provides a higher density of carboxyl groups compared to poly(acrylic acid) alone, enabling a more efficient conjugation of therapeutic and targeting moieties [21]. These carboxyl groups also exhibit pH-responsive behavior, undergoing protonation/deprotonation in response to environmental pH changes. This property is particularly valuable for targeted delivery to acidic tumor microenvironments (pH~6.5–6.8), where the PAM coating can undergo conformational changes that trigger drug release and/or enhance cellular uptake [22,23].

In this study, we investigate PAM-coated SPION clusters for combined magnetic targeting, hyperthermia, and pH-responsive drug delivery applications. We hypothesize that by controlling the clustering of PAM-coated SPIONs we may enhance their magnetic properties for improved magnetic targeting and hyperthermia efficiency. Furthermore, the carboxyl-rich coating will facilitate pH-responsive drug release in acidic tumor microenvironments and enable multi-modal functionalization. Our approach aims to address the current limitations of SPION-based systems by combining enhanced magnetic properties with a versatile surface chemistry for multi-modal applications.

Specifically, we demonstrate the following: (1) PAM-stabilized SPION clusters with controlled size distributions exhibit enhanced magnetic properties with a direct correlation between crystallite size, magnetic behavior, and functional performance; (2) the PAM coating provides excellent colloidal stability and pH-responsive behavior; (3) a dual targeting approach combining antibody conjugation with magnetic guidance significantly enhances cellular uptake; and (4) a novel alternating magnetic field (AMF) pre-treatment protocol dramatically improves therapeutic efficacy compared to conventional approaches. This integrated platform represents a significant advance in nanomedicine approaches for cancer therapy, with potential applications in the treatment of various solid tumors.

## 2. Results

### 2.1. Development and Characterization of SPION Clusters

#### 2.1.1. Controlled Synthesis and Size Distribution

The arrested precipitation method, with systematically varied synthetic parameters, successfully yielded three distinct magnetic nanocluster formulations (MNs1–3) with controlled size distributions. Dynamic light scattering measurements revealed well-defined populations with hydrodynamic diameters of 61.3 ± 8.1 nm, 80 ± 9.7 nm, and 100.2 ± 6.4 nm for MNs1, MNs2, and MNs3 respectively, all exhibiting narrow size distributions ((PDI < 0.2) (Table 1 & Figures S1 and S2)). This narrow size distribution is essential for predictable in vivo behavior and consistent performance [24,25]. TEM analysis confirmed that these size differences arise from the controlled clustering of primary SPION nanoparticles into progressively larger assemblies (Figures S3 and S4).

**Figure 1:**
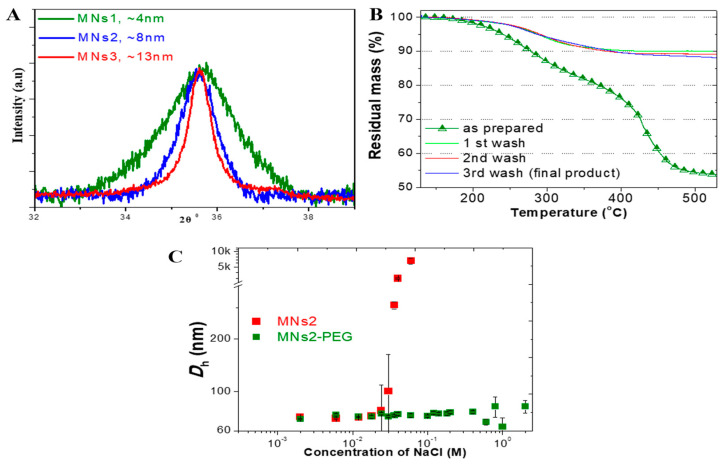
Characterization of MNs. (**A**) X-ray diffraction patterns demonstrating different crystallite sizes across formulations (4 nm, 8 nm, and 13 nm for MNs1, MNs2, and MNs3, respectively). (**B**) Thermogravimetric analysis showing stable polymer coating after multiple washing steps with approximately 10% organic content remaining. (**C**) Colloidal stability of PEGylated versus non-PEGylated formulations in increasing NaCl concentrations.

X-ray diffraction analysis revealed that small changes in the synthetic procedure markedly affected the nanocrystallite size of the products. These size differences are significant, because they directly influence the magnetic properties of the resulting nanoclusters, with larger crystallites generally exhibiting stronger magnetic responses [26]. The primary crystallite sizes were determined to be approximately 4 nm, 8 nm, and 13 nm for MNs1, MNs2, and MNs3, respectively (Figure 1A), allowing us to evaluate how crystallite size affects the performance of PAM-stabilized SPIONs across different applications.

#### 2.1.2. Surface Chemistry and Modification

The successful surface modification was confirmed through multiple complementary techniques. Thermogravimetric analysis demonstrated strong polymer binding, with approximately 10% stable organic content remaining after multiple washing steps (Figure 1B). This significant finding indicates that the poly(acrylic acid-co-maleic acid) coating forms robust bonds with the iron oxide surface that resist displacement even under extensive purification, a critical factor for in vivo applications.

Initial zeta potential measurements (Table 1) showed a highly negative surface charge (≈−50.2 mV), confirming the presence of numerous deprotonated carboxyl groups from the poly(acrylic acid-co-maleic acid) coating. These carboxyl groups serve dual functions: they provide electrostatic stabilization and serve as reactive sites for further functionalization.

The subsequent PEGylation was verified by ATR FT-IR spectroscopy through characteristic C-H vibrations at 2800–2900 cm^−1^ and evidence of the formation of amide bonds around 1698 cm^−1^, accompanied by a shift in zeta potential to =−10.9 mV (Figure S5). The reduction in negative charge results from the formation of amide bonds between PEG amine groups and PAM carboxylate groups, consuming the negatively charged carboxyl sites. Further antibody conjugation resulted in near-neutral zeta potential values, reflecting the addition of the protein component. Antibody conjugation achieved yields between 60% and 85% as determined by the Bradford microassay, demonstrating efficient utilization of the carboxyl groups for biomolecule attachment, consistent with previous reports.

The nanoclusters demonstrated a remarkable stability under various physiologically relevant conditions. While zeta potential magnitudes were below the traditional ±20 mV threshold, the PEGylated formulations maintained their colloidal stability through steric stabilization mechanisms in high ionic strength media, showing no significant size changes in NaCl concentrations up to 2 M (Figure 1C). This exceptional salt tolerance is critical for maintaining stability in biological fluids [27]. In contrast, the non-PEGylated formulations showed rapid aggregation under the same conditions, highlighting the importance of the PEG modification. Long-term stability studies revealed the maintenance of the initial size distribution over 30 days in physiological conditions for the PEGylated formulations, further confirming their suitability for biomedical applications (Figure S6).

#### 2.1.3. Magnetic Properties and Their Application-Specific Relevance

**Figure 2:**
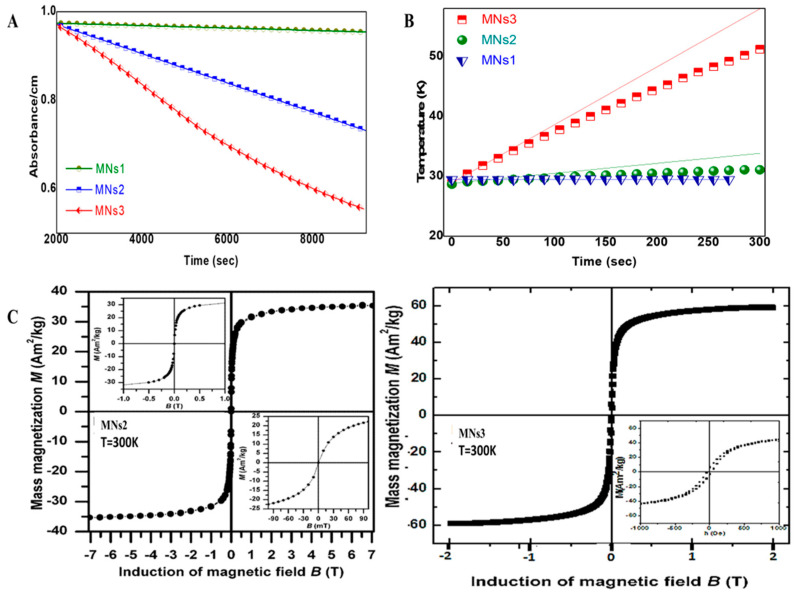
Magnetic properties of MNs. (**A**) Magnetophoresis profiles showing size-dependent magnetic response, with MNs3 exhibiting superior magnetic mobility. (**B**) Heating curves under alternating magnetic field conditions (50 Oe, 400 kHz) showing non-linear increase in heating efficiency from MNs1 (0 W/g) to MNs2 (6.7 W/g) to MNs3 (37.67 W/g). (**C**) Hysteresis loops at 300 K demonstrating superparamagnetic behavior for MNs2 and MNs3, with 68% higher magnetization for MNs3.

Magnetic characterization revealed a size-dependent enhancement of properties across the formulations. Magnetophoresis studies demonstrated that MNs3, composed of the largest nanocrystallites (~13 nm), displayed a superior magnetic response compared to smaller formulations (Figure 2A). This finding is particularly significant for magnetic targeting applications, where stronger response to external fields enables a more effective guidance to target tissues.

SQUID magnetometry measurements revealed size-dependent magnetic properties among the synthesized nanoparticle formulations. MNs2 exhibited a saturation magnetization of 35.31 Am^2^/kg with an apparent coercivity of 26.8 mT, while MNs3 demonstrated a higher saturation magnetization of 59 Am^2^/kg and reduced apparent coercivity of 3.2 mT (Figure 2B). When normalized for magnetic content as determined by TGA (84% and 86%, respectively), the effective magnetization values were calculated as 42 Am^2^/kg for MNs2 and 68.6 Am^2^/kg for MNs3. These values represent approximately 50–70% of the theoretical maximum for bulk magnetite (92–98 Am^2^/kg) [28], which is typical for superparamagnetic iron oxide nanoparticles due to surface spin canting effects. This indicates that the PAM coating preserves significant magnetic properties while providing surface functionality.

Mössbauer spectroscopy confirmed that both samples exhibit superparamagnetic behavior at room temperature, with spectral features typical of γ-Fe_2_O_3_ and/or Fe_3_O_4_ phases indicating Fe^3+^ as the dominant oxidation state, despite the different iron precursors used in synthesis (Fe^2+^ from FeSO_4_·7H_2_O for MNs2 and Fe^3+^ from FeCl_3_·6H_2_O for MNs3). The similar average blocking temperatures for both samples indicate comparable average particle sizes despite their different magnetic responses. This superparamagnetic property is essential for biomedical applications, as it prevents magnetic aggregation when the external field is removed [6].

Under alternating magnetic field conditions (50 Oe, 400 kHz), heating efficiency increased with cluster size, reaching specific absorption rate (SAR) values of 37.67 W/g for MNs3 (Figure 2C). This value, while not reaching the ideal threshold for clinical hyperthermia applications (typically > 100 W/g) [7], demonstrates a significant heat generation capacity that can be leveraged for triggered drug release and enhanced therapeutic effects through mild hyperthermia (39–41 °C). The observed size-dependent increase in SAR values aligns with theoretical predictions and confirms that controlling the nanocrystallite size via the PAM-stabilized clustering approach effectively modulates its magnetic heating properties.

MRI relaxometry demonstrated T_2_ contrast enhancement capabilities across all PEGylated MNs, with size-dependent variations in relaxivity values (Figure S7). The transverse relaxivity (r_2_) increased with nanocrystallite size, from 388.3 ± 12.5 s^−1^mM^−1^ for MNs2-PEG to 519.1 ± 32.9 s^−1^mM^−1^ for MNs3-PEG, while longitudinal relaxivity (r_1_) values were notably low (1.09 ± 0.02 and 0.52 ± 0.02 s^−1^mM^−1^, respectively). Consequently, the r_2_/r_1_ ratios for MNs2-PEG and MNs3-PEG were 356.23 and 998.21, respectively.

The exceptionally high r_2_/r_1_ ratios observed for MNs2-PEG and MNs3-PEG (356.23 and 998.21) suggest that these formulations would produce a strong negative contrast in T_2_-weighted images. For comparison, literature values at 3T of commercial agents like Ferumoxytol [29] show r_1_ = 10.0 ± 0.3 s^−1^mM^−1^ and r_2_ = 62.3 ± 1.7 s^−1^mM^−1^ (although at 7T r_1_ is expected to be lower). This size-dependent increase in r_2_/r_1_ ratio can be attributed to the clustering effect of the magnetic cores within the PAM matrix, which enhances local magnetic field inhomogeneities.

Based on the superior MRI contrast enhancement observed with MNs3-PEG compared to MNs2-PEG, this formulation was selected for all subsequent experiments. Therefore, from this point forward, MNs-PEG and MNs-PEG-OX26 refer specifically to the MNs3 formulation

### 2.2. Functional Capabilities: Targeting Efficiency and hCMEC/D3 Monolayer Transport

#### 2.2.1. Cellular Uptake Studies

**Figure 3:**
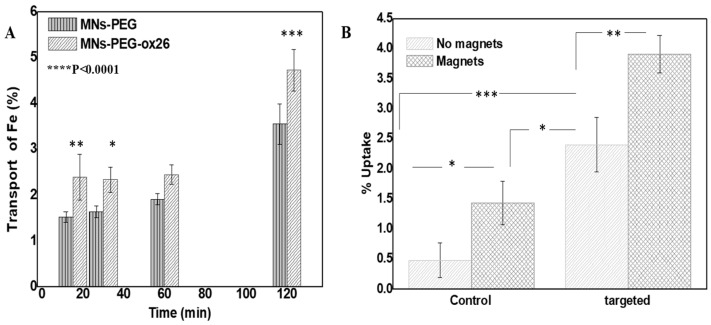
Targeting efficiency of targeted MNs-PEG. (**A**) Time-dependent transcytosis of PEGylated magnetic nanoparticles (MNs-PEG) with and without OX-26 antibody across an in vitro blood–brain barrier model (hCMEC/D3 monolayer). Fe transport (%) was measured at 15, 30, 60, and 120 min. Data are presented as mean ± SD (n ≥ 2). Error bars indicate standard deviation. (**B**) Quantitative cellular uptake in B16 melanoma cells comparing non-targeted (0.48 ± 0.28%), antibody-targeted (2.4 ± 0.45%), magnetic field-guided (1.4 ± 0.3%), and combined targeting approaches (3.9 ± 0.3%), demonstrating an 8-fold enhancement with dual targeting. Individual differences are marked by * for *p* ≤ 0.05, by ** for *p* ≤ 0.01, and by *** *p* ≤ 0.001.

Quantitative uptake analysis revealed a significant enhancement with combined targeting strategies. Non-targeted MNs-PEG showed a baseline uptake of 0.48 ± 0.28%, while antibody-conjugated formulations achieved 2.4 ± 0.45% uptake (*p* < 0.001). The application of a magnetic field further increased uptake to 3.9 ± 0.3% for antibody-targeted formulations, representing an 8-fold increase over non-targeted formulations without a magnetic field (*p* < 0.001) (Figure 3A). This substantial improvement demonstrates the potential clinical significance of the combined targeting approach, as enhanced cellular uptake is directly linked to improved therapeutic efficacy [15]. The combination of targeting and magnetic fields’ application resulted in a 3.92% ± 0.31% uptake, which was slightly higher than the 3.81% ± 0.57% expected from the additive effects (Bliss Independence Index = 1.03, 95% CI: 0.69–1.37). While this difference was not statistically significant (*p* = 0.81), these results indicate that the two approaches work additively rather than synergistically or antagonistically, allowing for predictable enhancement in targeting efficiency when combining antibody and magnetic approaches.

#### 2.2.2. hCMEC/D3 Monolayer Transport Studies

The transport of Fe across the hCMEC/D3 monolayer increased over time for both nanoparticle formulations (Figure 3B). In the PEGylated MNs without an antibody (MNs-PEG), Fe transport rose modestly from 1.53 ± 0.11% at 15 min to 1.64 ± 0.13% at 30 min and 1.91 ± 0.12% at 60 min, then jumped to 3.56 ± 0.44% at 120 min. In the OX26-conjugated MNs (MN-PEG-OX26), the initial transport was higher: 2.40 ± 0.49% at 15 min, followed by 2.34 ± 0.27% at 30 min and 2.45 ± 0.21% at 60 min, reaching 4.73 ± 0.45% at 120 min. Thus, at each time point the OX26-targeted particles delivered more Fe across the barrier (e.g., ~57% higher at 15 min and ~33% higher at 120 min than PEG-MNs), indicating the enhanced capability of MN-PEG-OX26 to target the transferrin receptor and permeate the monolayer.

### 2.3. Therapeutic Performance

#### 2.3.1. Drug Loading and Release Properties

The polymer-coated nanoclusters demonstrated a high drug loading capacity, achieving a 20% *w*/*w* doxorubicin loading efficiency. This substantial loading capacity is attributable to the numerous carboxyl groups in the PAM coating, which interact with the amine groups of doxorubicin through both electrostatic and hydrogen bonding interactions [20].

**Figure 4:**
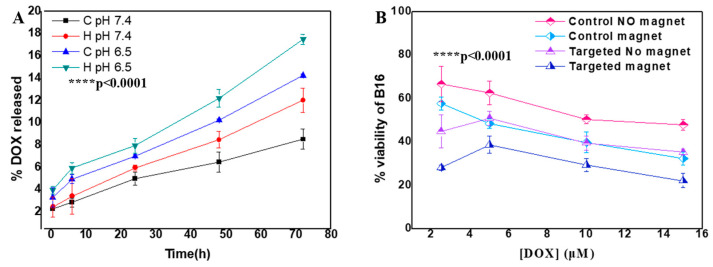
Drug loading and therapeutic performance. (**A**) Doxorubicin release profiles under different conditions (pH 7.4, pH 6.5, with (H) and without AMF application (C)) over 72 h, showing enhanced release at tumor-relevant pH (14.27 ± 0.13% at pH 6.5 vs. vs. 8.5 ± 0.9% at pH 7.4) and additional AMF-triggered release (17.5 ± 0.45%). (**B**) Cytotoxicity profiles of MNs-PEG (control) and MNs-PEG-OX26 (targeted) against B16 melanoma cells, showing enhanced efficacy with targeting and magnetic field application, resulting in 2.16-fold greater effectiveness at 15 μM compared to control without magnet. Two-way ANOVA *p* values of treatment type effect are reported.

Release studies revealed pH-dependent behavior, with enhanced release at pH 6.5 (14.27 ± 0.13%) compared to physiological pH 7.4 (8.5 ± 0.9%, *p* < 0.05) over 72 h (Figure 4A). This pH-sensitive release is particularly valuable for tumor targeting, as it enables preferential drug release in the acidic tumor microenvironment while minimizing premature release in circulation [21]. Application of an alternating magnetic field triggered an additional release (17.5 ± 0.45%, *p* < 0.05 vs. pH 6.5 alone), demonstrating the potential for externally controlled drug delivery.

The kinetic modeling of DOX’s release from PAM-coated SPION clusters showed that both zero-order and first-order models fit the data very well (R^2^ > 0.98), indicating a strong overall correlation between cumulative release and time (Figures S8 and S9). However, the Korsmeyer–Peppas model (Mt/M∞ = k·tn) provided a critical mechanistic insight: across all conditions—pH 7.4, pH 6.5, with and without an alternating magnetic field—the release exponent n ranged from 0.50 to 0.60 (Table 2). Such n values (0.5 < n < 1) are characteristic of anomalous (non-Fickian) transport, in which drug release is governed by a combination of diffusion and polymer relaxation [30].

This consistency in n confirms that neither an acidic pH nor magnetic actuation alters the fundamental release mechanism; instead, they accelerate the same anomalous process. We hypothesize that lowering the pH protonates PAM’s carboxyl groups, weakening electrostatic DOX binding, while magnetic field-induced nanoparticle vibrations enhance polymer chain mobility, both of which increase the rate of anomalous transport without changing its underlying nature.

#### 2.3.2. Anti-Proliferative Effects

Anti-proliferative studies demonstrated that MNs significantly enhance DOX’s cytotoxicity in B16 melanoma cells (Figure 4B). The application of a magnetic field significantly reduced cell viability in control groups at higher DOX concentrations (10 μM: *p* < 0.05; 15 μM: *p* < 0.01). Targeting alone significantly improved cytotoxicity at all concentrations (*p* < 0.05 to *p* < 0.01). The combination of targeting and magnetic field yielded the greatest enhancement in cytotoxicity across all concentrations (*p* < 0.01 to *p* < 0.001), resulting in a 2.16-fold greater effectiveness at 15 μM compared to the control without magnetic field application, demonstrating the added benefit of combining SPION-mediated targeting with magnetic field guidance.

#### 2.3.3. AMF-Enhanced Therapeutic Effects

**Figure 5:**
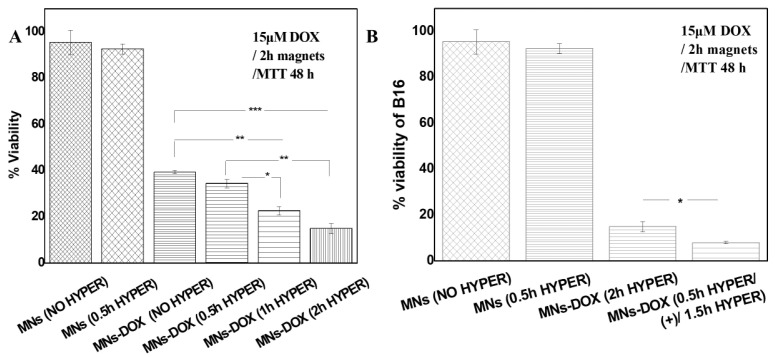
Enhanced therapeutic efficacy with optimized AMF protocols. (**A**) Cell viability after treatment with different AMF durations (0.5 h, 1 h, and 3 h) showing time-dependent enhancement of cytotoxicity: 39.39% (no AMF), 34.43% (0.5 h), 22.58% (1 h), and 14.92% (2 h). (**B**) Comparison of conventional (post-treatment) versus optimized (pre + post) AMF application protocols, demonstrating superior efficacy of pre-treatment approach (7.95% viability) compared to post-treatment only (14.92% viability), representing 87% improvement in therapeutic outcome. Two-way ANOVA *p* values of antibody effect are reported. Individual differences are marked by * for *p* ≤ 0.05, by ** for *p* ≤ 0.01, and by *** *p* ≤ 0.001.

The effects of an alternating magnetic field (AMF)’s application on the cytotoxicity of doxorubicin-loaded MNs-PEG (MNs-DOX) against B16 melanoma cells were investigated (Figure 5A). All experimental groups underwent a standardized 2 h incubation with nanoparticles in the presence of a permanent magnetic field to ensure consistent cellular uptake, followed by medium replacement before any AMF application.

Unloaded pegylated magnetic nanoparticles (MNs) exhibited a negligible cytotoxicity both without AMF exposure (95.44% viability) and with a 0.5 h AMF application (92.55% viability). This lack of significant cell death (*p* > 0.05) confirms that AMF exposure with our MN formulation was insufficient to cause direct cytotoxicity at these parameters.

Doxorubicin-loaded nanoparticles (MNs-DOX) reduced cell viability to 39.39% without the application of AMF, demonstrating effective drug delivery. When AMF was applied after MNs-DOX treatment, we observed a duration-dependent enhancement of cytotoxicity, with viabilities of 34.43% (0.5 h), 22.58% (1 h), and 14.92% (2 h). Statistical analysis revealed significant differences between these groups (*p* < 0.01), suggesting that AMF’s application enhances the cytotoxic effect of doxorubicin in a duration-dependent way.

#### 2.3.4. Superior Efficacy of AMF Pre-Treatment Protocol

A pre-treatment protocol was devised and performed to test AMF’s effect on cells (Figure 5B). Specifically, cells were exposed to AMF for 0.5 h prior to the addition of MNs-DOX, followed by 1.5 h AMF post-treatment. This resulted in a dramatic decrease in cell viability to 7.95%. This sequential protocol achieved a significantly higher cytotoxicity (*p* < 0.001) than the same total AMF duration (2 h) applied only after MNs-DOX treatment (14.92% viability). The superior efficacy of pre-treatment AMF suggests that initial exposure induces a cellular sensitization that enhances the subsequent drug action.

The comparative efficacy of different AMF application protocols revealed that the optimized protocol (0.5 h pre + 1.5 h post) achieved a 1.87-fold (87% improvement) greater cell death compared to the conventional post-treatment approach (Table 3). This finding represents a significant advance in maximizing the therapeutic efficacy of magnetic nanoparticle-based drug delivery systems.

## 3. Discussion

This study demonstrates that poly(acrylic acid-co-maleic acid)-stabilized SPION clusters represent a versatile platform for an integrated cancer diagnosis and therapy. The controlled synthesis approach yielded nanoclusters with a tunable size and magnetic properties, while the PAM coating provided excellent colloidal stability, pH-responsive behavior, and high drug loading capacity. The dual targeting approach combining antibody conjugation with magnetic guidance significantly enhanced cellular uptake and therapeutic efficacy, and the novel AMF pre-treatment protocol further improved treatment outcomes.

### 3.1. Enhanced Magnetic Properties Through Controlled Clustering

While Mössbauer spectroscopy confirmed that both MNs2 and MNs3 are superparamagnetic at room temperature, their magnetic responses differ significantly in ways that directly impact their functional performance. The 68% higher saturation magnetization observed in MNs3 compared to MNs2 can be attributed to three key factors: a higher content of magnetic material, larger magnetic nuclei, and optimized particle clustering. These structural differences, rather than fundamentally different magnetic states, are responsible for their dramatically different functional behaviors. The hysteresis loops measured at 300 K reflect these differences, with MNs2 displaying a wider apparent hysteresis with higher coercivity compared to MNs3′s narrower loop with minimal coercivity (Figure 2C). The superior magnetic response of MNs3 (13 nm crystallites) aligns with theoretical models that predict optimal magnetic susceptibility for crystallites between 12 and 20 nm due to reduced surface spin disorder and improved magnetic anisotropy stabilization [31].

The dramatic enhancement in heating efficiency observed for MNs3 (SAR: 37.67 W/g) compared to MNs2 (SAR: 6.7 W/g) reveals the non-linear relationship between crystallite size and functional performance (Figure 2A,B). This 5.6-fold increase in heating capacity with only modest increases in crystallite size suggests that there exists a critical threshold for optimal energy dissipation under alternating magnetic fields, likely related to the transition from multi-domain to single-domain magnetic behavior and the subsequent relaxation dynamics [32,33].

Similarly, the optimal MRI contrast properties—especially the exceptionally high r_2_/r_1_ ratios of 356.23 and 998.21 for MNs2-PEG and MNs3-PEG, respectively—represent a significant advancement over commercial contrast agents. The dramatic decrease in r_1_ values with increasing crystallite size, coupled with enhanced r_2_ relaxivity, creates ideal conditions for T_2_-weighted imaging applications. These values suggest potential advantages for detecting small lesions or subtle tissue abnormalities that might be missed using conventional contrast agents [34].

Our clustering approach effectively addresses a fundamental challenge in nanoparticle design: the need to balance enhanced magnetic properties (favored by larger particles) with colloidal stability (typically compromised in larger systems). By creating clusters of optimally sized crystallites within a protective PAM matrix, we maintain stability while amplifying their collective magnetic behavior. This strategy provides distinct advantages over both small individual particles (with a limited magnetic response) and large single particles (with a poor colloidal stability).

### 3.2. Advantages of PAM Coating for Multi-Functional Applications

Our results confirm that a PAM coating provides multiple advantages over conventional surface modifications. The high density of carboxyl groups enables both colloidal stabilization and pH-responsive behavior, with enhanced drug release (Figure 4A) at a tumor-relevant pH (6.5) compared to physiological conditions (7.4). This pH sensitivity is particularly valuable for preferential drug release in tumor microenvironments, potentially reducing systemic toxicity.

The kinetic analysis of drug release revealed consistent anomalous (non-Fickian) transport mechanisms across different conditions, suggesting that both a pH reduction and AMF application accelerate the same underlying release process rather than activating different pathways. This mechanistic understanding could guide the future optimization of stimulus-responsive delivery systems.

Furthermore, the carboxyl-rich PAM coating allowed efficient bioconjugation, with antibody attachment yields of 60–85%. This high conjugation efficiency is essential for effective active targeting and compares favorably with other nanoparticle surface modifications [35]. In fact, MNs-PEG-OX-26 showed consistently higher transport across the hCMEC/D3 monolayer than non-targeted particles, with the disparity becoming most pronounced at longer incubation times (Figure 3A).

Antibodies against the transferrin receptor are a well-established method for brain drug delivery, as they bind abundantly on brain capillaries. In fact, anti-TfR targeting has been shown to “dramatically increase” nanoparticle uptake into brain endothelial cells. The enhanced transport efficiency of MNs-PEG-OX-26 suggests that conjugating other payloads (such as neuroactive drugs or oligonucleotides) to this platform could improve their CNS bioavailability. This strategy has been demonstrated in vivo—for example, OX26-functionalized liposomes showed a markedly higher brain accumulation than non-targeted formulations [36].

### 3.3. Synergistic Benefits of Dual Targeting Approach

The combination of antibody targeting and magnetic guidance demonstrated additive benefits for cellular uptake, increasing internalization 8-fold compared to non-targeted formulations (Figure 3B). This significant enhancement translates directly to improved therapeutic outcomes, with dual-targeted doxorubicin-loaded nanoclusters achieving a 2.16-fold greater cytotoxicity than non-targeted equivalents at equivalent drug concentrations (Figure 4B). Similar results were reported with dual-targeted doxorubicin-loaded magnetic liposomes [37].

The additive nature of these targeting mechanisms (Bliss Independence Index = 1.03) suggests that they operate through complementary pathways: antibody targeting likely enhances receptor-mediated endocytosis, while magnetic guidance increases particle concentration at the cell surface. This complementarity offers flexibility in clinical applications, as either approach could be emphasized depending on tumors’ characteristics and their accessibility to external magnetic fields.

### 3.4. Novel Insights into AMF Pre-Treatment Effects

The most striking finding of our study is the superior efficacy of AMF pre-treatment before the application of drug-loaded nanoparticles. This sequential protocol achieved an 87% greater cell death compared to conventional post-treatment application, despite using identical AMF parameters and exposure durations (Figure 5B). This unexpected enhancement suggests that AMF exposure initiates cellular changes that sensitize cancer cells to subsequent chemotherapy.

The superior efficacy of this AMF pre-treatment protocol could be attributed to complementary mechanisms. They likely include the following: (1) induction of sub-lethal oxidative stress that sensitizes cells to doxorubicin’s ROS-generating effects [38]; (2) increased membrane permeability through temporary pore formation or an altered membrane potential, enhancing doxorubicin’s uptake [39]; (3) disruption of calcium and other ions’ homeostasis that primes apoptotic pathways [40]; and (4) activation of stress-response signaling or epigenetic modifications that favor cell death when subsequently challenged with doxorubicin.

These changes, while insufficient to independently trigger cell death (as evidenced by the absence of cytotoxicity in AMF-treated unloaded MNs), effectively “prime” cancer cells to become more susceptible to chemotherapy. This sensitization phenomenon aligns with previous reports that pulsing magnetic fields can enhance antitumor drugs’ efficacy even without nanoparticle carriers [41], suggesting that electromagnetic field exposure may directly alter cellular susceptibility to chemotherapeutic agents.

### 3.5. Clinical Translation Potential

Several aspects of our PAM-SPION nanoclusters make them promising candidates for clinical translation. Their synthesis method is scalable and reproducible, using biocompatible components with established safety profiles. Iron oxide nanoparticles are already FDA-approved for various applications, and poly(acrylic acid-co-maleic acid) has demonstrated excellent biocompatibility in previous studies [18,19].

The dual targeting capability offers flexibility in clinical applications, potentially addressing challenges in reaching specific tumor types. For example, brain tumors might benefit from the combination of transferrin receptor targeting (via the OX-26 antibody) and magnetic guidance to overcome blood–brain barrier limitations. Similarly, the pH-responsive behavior could enhance specificity for solid tumors with acidic microenvironments while reducing systemic toxicity.

The most clinically significant finding—the enhanced efficacy of AMF pre-treatment—offers a potential paradigm shift in how magnetic field-based therapies are applied. This approach could enable more effective treatments without increasing the nanoparticle dose or magnetic field intensity, both of which are limited by safety considerations in clinical settings.

### 3.6. Study Limitations and Future Directions

While these results are promising, several limitations should be acknowledged. The pH-responsive drug release profile, though measurable, shows a modest selectivity between physiological and acidic conditions, suggesting the need for optimization to enhance therapeutic specificity. Cellular uptake efficiency, while demonstrating targeting capability, could be improved through further surface modifications or the optimization of targeting ligands. Additionally, comprehensive in vivo biocompatibility and pharmacokinetic studies are essential before clinical translation, particularly to evaluate long-term tissue distribution and clearance. Future work should focus on optimizing the pH-responsive mechanism, enhancing cellular internalization, and conducting systematic toxicity evaluations in relevant animal models.

## 4. Materials and Methods

### 4.1. Materials

FeSO_4_ × 7H_2_O (Lab NV), FeCl_3_ × 6H_2_O (Sigma-Aldrich, Saint Louis, MO, USA), Poly(acrylic acid-co-maleic acid) solution 3 kDa (50% solution at 25 °C, Sigma-Aldrich), methoxy PEG amine 2 kDa (Creative PEGWorks, Durham, NC, USA), Maleimide PEG amine 2 kDa (Creative PEGWorks), 1-Ethyl-3-(3-dimethylaminopropyl) carbodiimide (EDC) (Sigma-Aldrich), N-Hydroxysulfosuccinimide sodium salt (S-NHS) (Sigma-Aldrich), HCl (Carlo Erba, Cornaredo, Italy), NaCl (Merck Chemicals, Boston, MA, USA), NaOH (Merck Chemicals), Doxorubicin Hydrochloride (Tocris Bioscience, Bristol, UK), and Thiolated OX-26 (Serotec, Oxford, UK) were used as received. Amicon Ultra-15 Centrifugal Filter Devices (10 kDa and 30 kDa cut-off, (Merck KGaA, Darmstadt, Germany)) were used for samples’ concentration and purification. Protein concentrations were measured using Bradford microassay reagent (Bio-Rad, Hercules, CA, USA). Traut’s reagent (2-iminothiolane hydrochloride) was purchased from Pierce (Rockford, IL, USA). Ellman’s reagent (5,5′-dithiobis-(2-nitrobenzoic acid), DTNB) was obtained from Sigma-Aldrich. Borate buffer (50 mM, pH 8.5) and phosphate-buffered saline (PBS, pH 7.4) were prepared using standard protocols. All other chemicals were of analytical grade and were obtained from either Sigma-Aldrich or Merck.

### 4.2. Synthesis and Characterization

#### 4.2.1. Synthesis of PAM-SPION Nanoclusters

The arrested precipitation method of a single ferrous precursor was used.

##### General Synthesis Procedure

In a typical synthesis, poly(acrylic acid-co-maleic acid) (PAA-co-MA, 0.15 mg) was dissolved in distilled water (30 mL) in a round-bottom flask equipped with a mechanical stirrer. Separately, the iron precursor was dissolved in distilled water (10 mL) containing 37% HCl (60 μL). The iron solution was added to the polymer solution under heating at 40 °C, with continuous mechanical stirring at 600 rpm. The appropriate base solution was then added dropwise at 40 °C, and the reaction mixture was heated to the specified maximum temperature while maintaining constant stirring. The reaction was allowed to proceed for the designated time period.

##### Specific Formulations

MNs1 (PAA-co-MA/FeSO_4_/NH_3_): Iron(II) sulfate heptahydrate (FeSO_4_·7H_2_O, 0.72 g) was used as the iron precursor. Ammonium hydroxide (NH_3_, 30%, 4 mL) was added as the precipitating agent. The reaction was conducted at a maximum temperature of 70 °C for 3 h and 40 min.

MNs2 (PAA-co-MA/FeSO_4_/NaOH): Iron(II) sulfate heptahydrate (FeSO_4_·7H_2_O, 0.72 g) was used as the iron precursor. Sodium hydroxide (NaOH, 1 M, 10 mL) was added as the precipitating agent. The reaction was conducted at a maximum temperature of 50 °C for 1 h and 40 min.

MNs3 (PAA-co-MA/FeCl_3_/NaOH): Iron(III) chloride hexahydrate (FeCl_3_·6H_2_O, 0.328 g) was used as the iron precursor under higher pH conditions. Sodium hydroxide (NaOH, 1 M, 15 mL) was added as the precipitating agent. The reaction was conducted at a maximum temperature of 60 °C for 1 h and 40 min.

##### Purification and Final Processing

Following synthesis, all products were purified by successive centrifugations to remove undesirable residuals and byproducts. Three centrifugation cycles were performed until the supernatant conductivity matched that of triple-distilled water, indicating the complete removal of ionic impurities. The purified colloidal suspensions were transferred to glass vials and sonicated for 15 min to ensure homogeneous dispersion. A final centrifugation step was performed for 20 min at approximately 4000× *g* to remove aggregates and ensure a narrow size distribution. The final magnetic nanoparticle suspensions were obtained at a concentration of 1% *w*/*v* (calculated as Fe_2_O_3_ equivalent, assuming Fe_2_O_3_).

#### 4.2.2. PEGylation and Antibody Conjugation

Conjugation of the outer carboxyl groups of PAM with amine-terminated poly(ethylene glycol) was accomplished using EDC and S-NHS chemistry. According to the molar content of the free carboxylic groups of the PAM coating, an optimized ratio of 1:0.1 EDC:S-NHS was used to react with 0.08 parts of mPEG-NH_2_. This lower S-NHS ratio was found to provide an optimal conjugation yield while minimizing hydrolysis and side reactions. For further functionalization, a molar ratio of 1:3 MAL-PEG-NH_2_:mPEG-NH_2_ was used to maintain the same total moles of PEG. A protocol of three successive centrifugations at 5000× *g* for 20 min at 4 °C was used to purify the product.

For antibody conjugation, the anti-transferrin receptor-specific monoclonal antibody (MAb) OX-26 was immobilized by thiol–maleimide covalent coupling. Free thiol groups were generated by reacting the antibody with Traut’s reagent in 0.15 M Na–borate buffer with 0.1 mM EDTA, pH 8.5. After incubation (90 min, 25 °C, under N_2_), the solution was concentrated and the buffer exchanged with PBS (pH 7.4) using an Amicon filter device (10 kDa cut-off at 4000× *g* for 15 min at 4 °C).

Ellman’s reagent [42] determined the number of sulfhydryl groups generated, using acetylcysteine for calibration. Using an OX-26–iminothiolane molar ratio of 1:10, an average of 3 primary amines per antibody were thiolated. Thiolated OX-26 was incubated with MNs-PEG-MAL overnight at 25 °C (molar ratio OX-26/MAL of 1:25). To remove unbound OX-26, the sample was ultracentrifuged at 30,000× *g* for 1 h at 4 °C. The conjugation yield was determined by measuring the unbound OX-26 concentration in the ultracentrifugation supernatant using the Bradford microassay.

#### 4.2.3. Physicochemical Characterization

Detailed methods for physicochemical characterization, including iron content determination, dynamic light scattering, zeta potential measurement, X-ray diffraction, thermogravimetric analysis, and infrared spectroscopy, are provided in Appendix A.

#### 4.2.4. Magnetic Characterization

Magnetic response was evaluated using a permanent Nd-Fe-B hand magnet. Magnetophoresis experiments were performed using a Hitachi (Tokyo, Japan) Digilab U-2800 spectrophotometer with a cylindrical Nd−Fe−B magnet placed next to the cuvette holder. Hyperthermia experiments were conducted on 1% *w*(Fe_2_O_3_)/*v* dispersions at a field of 50 Oe (4 kA m^−1^) and at 400 kHz. SQUID magnetization measurements were carried out using a Lakeshore (Carson, CA, USA) 7300 Vibrating Sample Magnetometer at 300 K. MRI relaxometry was performed using a 7.0 T Bruker Biospec 70/30 USR nuclear magnetic resonance spectrometer (Billerica, MA, USA), with detailed protocols provided in the Supporting Information.

### 4.3. Functional Studies

#### 4.3.1. Doxorubicin Loading and Release

A dispersion of MNs (120 μg Fe_2_O_3_) was mixed with doxorubicin hydrochloride in PBS (pH 7.40) at Fe_2_O_3_/DOX ratios between 0.1 and 0.5 *w*/*w* and shaken overnight at 37 °C. After centrifugation (30,000× *g*, 1 h), non-absorbed DOX in the supernatant was quantified by fluorescence spectroscopy (excitation 470 nm, emission 550 nm). The pellet was redispersed for further experiments. Loading efficiency was expressed as μg(DOX)/(μg(DOX) + μg(carrier)).

Release experiments were performed by redispersing the pellet in PBS (pH 6.5 or 7.4) at 37 °C. Hyperthermia’s effect on drug release was determined by applying an alternating magnetic field (same conditions as above) for 15 min before each sampling point. Released DOX was quantified by fluorescence spectroscopy.

#### 4.3.2. Cell Culture Studies

Human brain capillary endothelial cells (hCMEC/D3, passages 25–35) and B16 melanoma cells were cultured according to established protocols (detailed in Supporting Information). MTT assays evaluated cytotoxicity after 2 h and 24 h incubation with various nanoparticle formulations. Cell uptake studies measured the internalization of control MNs-PEG and MNs-PEG-OX26 using atomic absorption spectroscopy after appropriate cell processing. Blood–brain barrier (BBB) targeting studies were performed, according to a method previously reported by our lab [43], utilizing a hCMEC/D3 monolayer, for MNs-PEG-OX26 and control MNs-PEG.

The impact of AMF on cellular uptake and therapeutic efficacy was evaluated using multiple protocols: (1) Conventional post-treatment: cells were incubated with drug-loaded nanoparticles for 2 h, followed by 2 h AMF exposure. (2) Sequential treatment: cells were exposed to AMF for 0.5 h, then incubated with drug-loaded nanoparticles, followed by an additional 1.5 h AMF exposure. (3) Various control conditions including AMF-only, nanoparticle-only, and split AMF exposure without nanoparticles. All experiments were performed in triplicate with standardized cell counts, nanoparticle concentrations, and AMF parameters (50 Oe, 400 kHz).

### 4.4. Statistical Analysis

All experiments were performed in at least triplicate, and data are presented as mean ± standard deviation. Statistical significance was determined using one-way ANOVA, followed by Tukey’s post-hoc test for multiple comparisons. A two-tailed Student’s *t*-test was used for paired comparisons. Differences were considered statistically significant at *p* < 0.05. GraphPad Prism 9.0 software (GraphPad Software, Boston, MA, USA) was used for all statistical analyses.

## 5. Conclusions

In this study, a novel theranostic platform based on poly(acrylic acid-co-maleic acid)-stabilized superparamagnetic iron oxide nanoclusters was developed, which integrates magnetic targeting, hyperthermia, and pH-responsive drug delivery capabilities.

The results demonstrate the potential of these engineered PAM-SPION nanoclusters as a versatile platform for integrated cancer diagnosis and therapy. The combination of exceptional MRI contrast properties (r_2_/r_1_ ratios up to 998.21), efficient heating capabilities, and an enhanced drug delivery through dual targeting and an AMF-responsive release addresses the current limitations in nanomedicine approaches. The unexpected synergistic effect of AMF pre-treatment offers a promising new strategy for enhancing therapeutic outcomes without increasing drug or nanoparticle doses.

The clinical translation potential of this platform is significant, particularly for solid tumors with acidic microenvironments and tumors requiring an enhanced penetration of therapeutic agents, such as brain tumors and metastatic lesions. Future work will focus on an in vivo validation of these findings and further optimization of the AMF pre-treatment protocol for maximum therapeutic benefit with minimal side effects.

## Figures and Tables

**Table 1:** Size distribution by dynamic light scattering (intensity-based) of MNs1, MNs2, and MNs3 and the respective MNs conjugated with PEG (MNs-PEG) or PEG and OX-26 (MNs-PEG-OX26), showing controlled hydrodynamic diameters.

Sample	D_h_ (nm)	ζp (mV)
MNs1	61.3 ± 8.1	−49.3 ± 7.6
MNs2	80 ± 9.7	−48.1 ± 8.5
MNs3	100.2 ± 6.4	−50.2 ± 5.3
MNs2-PEG	97.3 ± 7.2	−11.2 ± 4.8
MNs3-PEG	112.4 ± 10.8	−10.9 ± 3.3
MNs2-PEG-OX26	129.2 ± 4.8	1.1 ± 0.8
MNs3-PEG-OX26	140.2 ± 8.4	1.96 ± 0.92

**Table 2:** Kinetic parameters and correlation coefficients (R^2^) for different kinetic models of doxorubicin release under different pH (7.4 or 6.5) and hyperthermia treatment or no hyperthermia (control).

Model	Parameter	Control pH 7.4	Hyperthermia pH 7.4	Control pH 6.5	Hyperthermia pH 6.5
Zero Order	Slope	0.086 ± 0.005	0.130 ± 0.005	0.146 ± 0.007	0.180 ± 0.011
	Intercept	2.47 ± 0.22	2.57 ± 0.19	3.60 ± 0.28	4.11 ± 0.45
	R^2^	0.988	0.996	0.993	0.989
First Order	Slope	−0.00091 ± 0.00005	−0.00140 ± 0.00005	−0.00160 ± 0.00008	−0.00202 ± 0.00014
	Intercept	4.58 ± 0.002	4.58 ± 0.002	4.57 ± 0.003	4.56 ± 0.006
	R^2^	0.990	0.996	0.992	0.986
Higuchi	Slope	0.80 ± 0.08	1.20 ± 0.15	1.34 ± 0.18	1.64 ± 0.27
	Intercept	1.30 ± 0.42	0.87 ± 0.83	1.70 ± 0.99	1.83 ± 1.47
	R^2^	0.973	0.954	0.948	0.926
Korsmeyer–Peppas	Slope (n)	0.51 ± 0.06	0.61 ± 0.06	0.54 ± 0.06	0.54 ± 0.06
	Intercept (log k)	0.38 ± 0.08	0.42 ± 0.08	0.55 ± 0.07	0.63 ± 0.08
	R^2^	0.889	0.899	0.907	0.884

**Table 3:** Comparative efficacy of 2 h post-treatment and 0.5 h pre- + 1.5 h post-treatment AMF application protocols.

Protocol	AMF Timing	Cell Viability (%)	Cell Death (%)	Relative Efficacy
Conventional	2 h post-treatment	14.92 ± 1.2	85.08	1.00 (baseline)
Optimized	0.5 h pre + 1.5 h post	7.95 ± 0.8	92.05	1.87 (87% improvement)

## Data Availability

The original contributions presented in this study are included in the article/Supplementary Materials. Further inquiries can be directed to the corresponding author.

## Supplementary Materials

The following supporting information can be downloaded at: https://www.mdpi.com/article/10.3390/molecules30132785/s1, Figure S1: DLS size distribution curves of PAM-coated nanoclusters. (A) MNs1, (B) MNs2, (C) MNs3, showing progression in hydrodynamic diameter and narrow size distributions (PDI < 0.2). Curves are representative of triplicate measurements. Figure S2: DLS size distribution curves of functionalized nanoclusters. (A) MNs2-PEG, (B) MNs3-PEG, (C) MNs2-PEG-OX26, (D) MNs3-PEG-OX26, demonstrating maintained narrow size distributions after surface functionalization. Curves are representative of triplicate measurements. Figure S3: TEM morphological characterization of PAM-coated nanoclusters. (A) MNs1, (B) MNs2, and (C) MNs3, demonstrating systematic progression in cluster size and organization. Scale bars = 50 nm. Figure S4: Higher magnification TEM analysis of nanocluster internal structure. (A) MNs2 and (B) MNs3 showing discrete primary SPION nanoparticles assembled within cluster frameworks, confirming the formation of multi-particle clusters. Scale bars = 20 nm. Figure S5: Characterization of MNs2-PEG. ATR FT-IR spectrum confirming the conjugation by the appearance of the PEG’s C-H vibrations at ~2800–2900 cm^−1^; Figure S6: Stability study of MNs2-PEG. Dynamic light scattering studies were performed for 30 days in order to compare the long-term colloidal behavior of MN products before and after PEGylation. PEGylated nanocrystallites remain stable even after 30 days, in contrast to the ones without PEG, which gradually aggregate and precipitate. Figure S7: Linear regression analysis of (A,C) longitudinal relaxivity (r_1_) and (B,D) transverse relaxivity (r_2_) for MNs2-PEG and MNs3-PEG formulations measured at 7T in phantom agar. Error bars represent standard deviation from T_1_/T_2_ curve fitting. Regression parameters are shown in each panel. Supplementary Methods: Detailed protocols for cell culture, MTT viability assays, and cellular uptake quantification. Figure S8: First-order kinetics linearization plots for release of DOX. Linear regression analysis showing ln(100 − % released) vs. time for (A) Control pH 7.4, (B) Control pH 6.5, (C) Hyperthermia pH 7.4, (D) Hyperthermia pH 6.5. High correlation coefficients (R^2^ > 0.98) confirm first-order release behavior across all conditions. Data points represent mean ± SD (n = 3); Figure S9: Zero-order kinetics linearization plots for release of DOX. Linear regression analysis showing % drug released vs. time for (A) Control pH 7.4, (B) Control pH 6.5, (C) Hyperthermia pH 7.4, (D) Hyperthermia pH 6.5. All conditions demonstrate an excellent linear fit (R^2^ > 0.98), indicating zero-order release kinetics. Data points represent mean ± SD (n = 3).

## Abbreviations

The following abbreviations are used in this manuscript:

AMF	Alternating magnetic field
BBB	Blood–brain barrier
DOX	Doxorubicin
EDC	1-Ethyl-3-(3-dimethylaminopropyl) carbodiimide
MNs	PAM-stabilized SPIONs
MNs-PEG:	PEGylated formulations
MNs-PEG-OX26:	Antibody-conjugated final formulations
PAM	Poly(acrylic acid-co-maleic acid)
PDI	Polydispersity index
PEG	Poly(oxyethylene)
ROS	Reactive oxygen species
SAR	Specific absorption rate
S-NHS	N-Hydroxysulfosuccinimide sodium salt
SPIONs	Superparamagnetic iron oxide nanoparticles

## Appendix A

Physicochemical characterization of MNs.

Spectrophotometric determination of Fe ions: The Fe_2_O_3_ content in the magnetic colloids was determined spectrophotometrically following the 1,10-phenanthroline method. The colloid (20 μL) was mixed in an 1.5 mL microcentrifuge tube with concentrated HCl 37% (40 μL) in order to dissolve the Fe_2_O_3_. After 30 min the sample had turned yellow and was transferred in a 10 mL volumetric flask. Hydroxylamine hydrochloride (NH_2_OH × HCl) was added (0.1 mL, 1 g L^−1^) in order to reduce the Fe^+3^ to Fe^+2^. 1,10-phenanthroline (1 mL, 100 g L^−1^) and CH_3_COONa (0.8 mL, 1.2 M).

Dynamic light scattering (DLS) was performed on aqueous dispersions of ∼0.01% *w*/*v* in Fe_2_O_3_ in pH 7.4. Electrokinetic measurements for the determination of the mobility and zeta potential (ζp) values of the suspensions were performed with a Malvern Instrument ZetaSizer Nano and with a 4 mW He−Ne laser, operating at a wavelength of 633 nm. DLS was performed using the same instrument, where scattered light was collected at a fixed angle of 173°. The hydrodynamic diameters (Dh) reported are the mean of three measurements, and each measurement was the sum of 12 correlograms and fitting procedures. The cumulants analysis was applied, and the Dh average values reported are the z-average mean Dh, unless otherwise stated. The reported polydispersity index (PdI) values, ranging between 0 for an ideally monodispersed sample and 1 for a system with a very broad size distribution, have been derived from the formula PdI = σ2 /Dh 2, where σ is the standard deviation of the distribution in nanometers. Zeta potential was measured for the same samples at 25 °C, by the same instrument (utilizing the Doppler electrophoresis technique). In some cases, the size distribution was measured during extended time periods of incubation for the estimation of MNs/MNs-PEG’s colloidal stability (up to 30 days). Additionally, MNs’ resistance to aggregation induced by increasing concentrations of NaCl was evaluated; MN samples (50 mm^3^) were mixed with aqueous NaCl solutions (950 mm^3^) of different concentrations (range 0–2 M), and the mixtures were incubated (37 °C). After 24 h, the size characteristics of the MNs were determined by DLS.

PEG binding on the polymeric corona was determined by a UV spectrometry method based on the absorbance shift of the complex formed between PEG and Lugol’s reagent. Briefly, samples were digested by the addition of 100 μL HCl 37%, followed by heating at 80 °C. Then, they were left to reach room temperature, and 25 μL of Lugol’s reagent was added. After the complex’s formation (approx.15 min), the absorbance was measured at 535 nm.

X-ray diffraction (XRD) was performed on a D-800 Siemens diffractometer, with Ni-filtered Cu Kα radiation. Samples were dried on a glass plate and then collected and ground. Ground samples were spread with the aid of ethanol on a Bruker sample holder with a Si wafer for low sample volumes.

Samples for TEM were prepared by casting a droplet of a dilute aqueous suspension (0.01% *w*/*v* in Fe_2_O_3_) of the hybrids on copper grids coated by Formvar carbon film. Micrographs were obtained by a JEOL, JEM-2100 instrument operating at 200 kV.

Thermogravimetric measurements (TA Instruments, Q500) were conducted in order to determine the polymer content of each sample. Measurements were performed under N_2_ flow. Neat P(MAA-g-EGMA) polymer was first studied in order to determine the residue of the polymer. Based on these results, the polymer content in the hybrids was finally determined.

In order to declare the existence of the amidic bond in MNs-PEG, an ATR study was performed, on a Nicolet 850FT-IR Spectrometer Rapid (Thermo Fisher Scientific, Waltham, MA, USA) and step scan ATR accessory Grazing angle reflectance accessory.

Relaxivity measurements were performed with a 7T Bruker Biospec 70/30 USR spectrometer at room temperature. Nanoparticles were dispersed in phantom agar at physiological pH at Fe concentrations of 0, 0.0313, 0.0625, 0.125, and 0.25, and 0.5 mM. T_1_, T_2_, and T_2_* relaxation times were measured using standard relaxometry sequences (RARE-VTR, RARE, and FLASH, respectively). Relaxivities (r_1_, r_2_, r_2_*) were calculated from the slope of the linear regression of 1/T_i_ versus Fe concentration.
