# Supplementary material for: Engineered PAM-SPION Nanoclusters for Enhanced Cancer Therapy: Integrating Magnetic Targeting with pH-Responsive Drug Release
Source: Molecules. 2025 Jun 28;30(13):2785. doi: 10.3390/molecules30132785 (PMC12250690; doi:10.3390/molecules30132785)
Supplement: Supplementary file 1 [file molecules-30-02785-s001.zip › molecules-3701062-supplementary.pdf]

Supplementary Data for:

Engineered PAM-SPION Nanoclusters for Enhanced Cancer Therapy: Integrating Magnetic Targeting with pH-Responsive Drug Release

Methods

Experiments on hCMEC/d3 cells. Immortalized human brain capillary endothelial cells (hCMEC/D3) (passage 25-35) were used. The cell line was obtained under license from Institut national de la Sante et de la Recherche Medicale (INSERM, Paris, France). Cells were seeded at 27,000 cells/cm^2 and grown in EBM-2 medium (Lonza, Basel, Switzerland) supplemented with 10 mM HEPES, 1 ng/ml basic FGF (bFGF), 1.4 μM hydrocortisone, 5 μg/ml ascorbic acid, penicillin-streptomycin, chemically defined lipid concentrate, and 5% ultralow IgG FBS. The cells were cultured at 37°C, 5% CO_2 / saturated humidity. All cultureware were coated with 0.1 mg/ml rat tail collagen type I (BD Biosciences). Medium was changed every 2-3 days.

Experiments on B16 cells. B16 melanoma cells were used. Cells were seeded at 27,000 cells/cm^2 and grown in RPMI medium (Gibco) supplemented with penicillin-streptomycin, and 10% ultralow IgG FBS. The cells were cultured at 37°C, 5% CO_2 / saturated humidity. Medium was changed every 2-3 days.

Cytotoxicity studies (MTT assay). Cells were grown on 96-well plates until confluent. Medium was replaced and the MNs (at concentrations used in the cell interaction studies and other concentrations) were incubated with cells for 2h and 24 h at 37°C, (5% CO_2 /saturated humidity). After incubation medium was removed and the cells were washed with PBS. Fresh medium containing 0.5 mg/ml 3-(4,5-Dimethylthiazol-2-yl)-2,5-diphenyltetrazolium bromide (MTT) was added. Cells were incubated for 4 h, the medium was removed and DMSO was added (at 37°C for 30 min) to dissolve the formazan crystals that formed. Alive cells (%) were calculated based on the formula (A570sample-A570background) / (A570control-A570background) x 100, where A570control is the OD-570 nm of untreated cells, and A570background the OD-570 nm of MTT without cells.

Cell uptake studies. For PEG-MNs uptake by cells, control PEG-MNs and OX26-PEG-MNs were incubated with confluent monolayers of hCMEC/D3 or B16 cells (1mg and 0,1mg of Fe/10^6 cells) in medium (containing 10% (v/v) FCS) at 37°C, for 60 min, then washed in ice-cold PBS (x3), detached from plates, re-suspended in PBS (after cell lysis in 2% Triton X-100). MNs uptake was estimated by Atomic Absorption. Cell auto absorption was always subtracted.

A

			Size (d.nm):	% Intensity:	St Dev (d.nm):
Z-Average (d.nm):	60,67	Peak 1:	73,52	100,0	36,58
PdI:	0,182	Peak 2:	0,000	0,0	0,000
Intercept:	0,963	Peak 3:	0,000	0,0	0,000
Result quality :	Good				

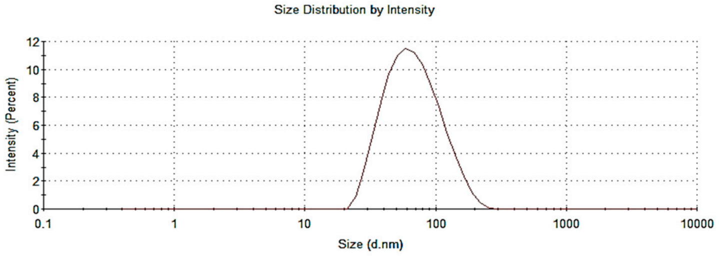

			Size (d.nm):	% Intensity:	St Dev (d.nm):
Z-Average (d.nm):	79,80	Peak 1:	92,74	100,0	36,27
PdI:	0,135	Peak 2:	0,000	0,0	0,000
Intercept:	0,919	Peak 3:	0,000	0,0	0,000
Result quality :	Good				

B

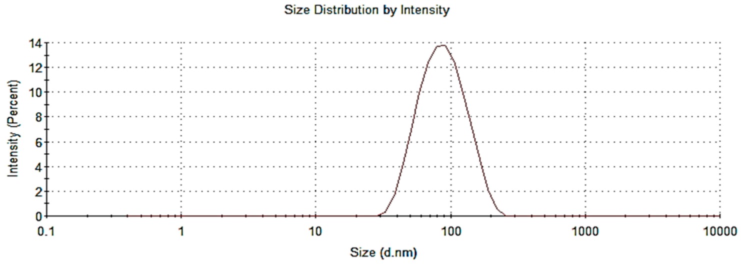

			Size (d.nm):	% Intensity:	St Dev (d.nm):
Z-Average (d.nm):	100,3	Peak 1:	113,5	100,0	45,65
PdI:	0,163	Peak 2:	0,000	0,0	0,000
Intercept:	0,925	Peak 3:	0,000	0,0	0,000
Result quality :	Good				

C

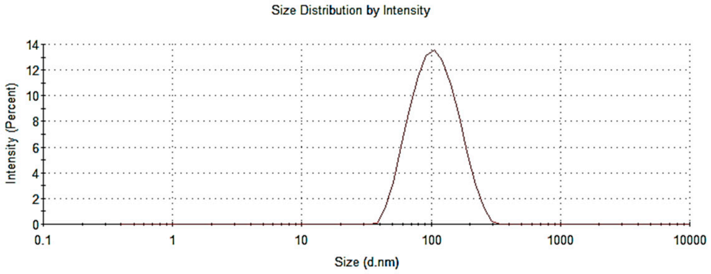

Figure S1. DLS size distribution curves of PAM-coated nanoclusters. (A) MNs1, (B) MNs2, (C) MNs3 showing progression in hydrodynamic diameter and narrow size distributions (PDI < 0.2). Curves are representative of triplicate measurements.

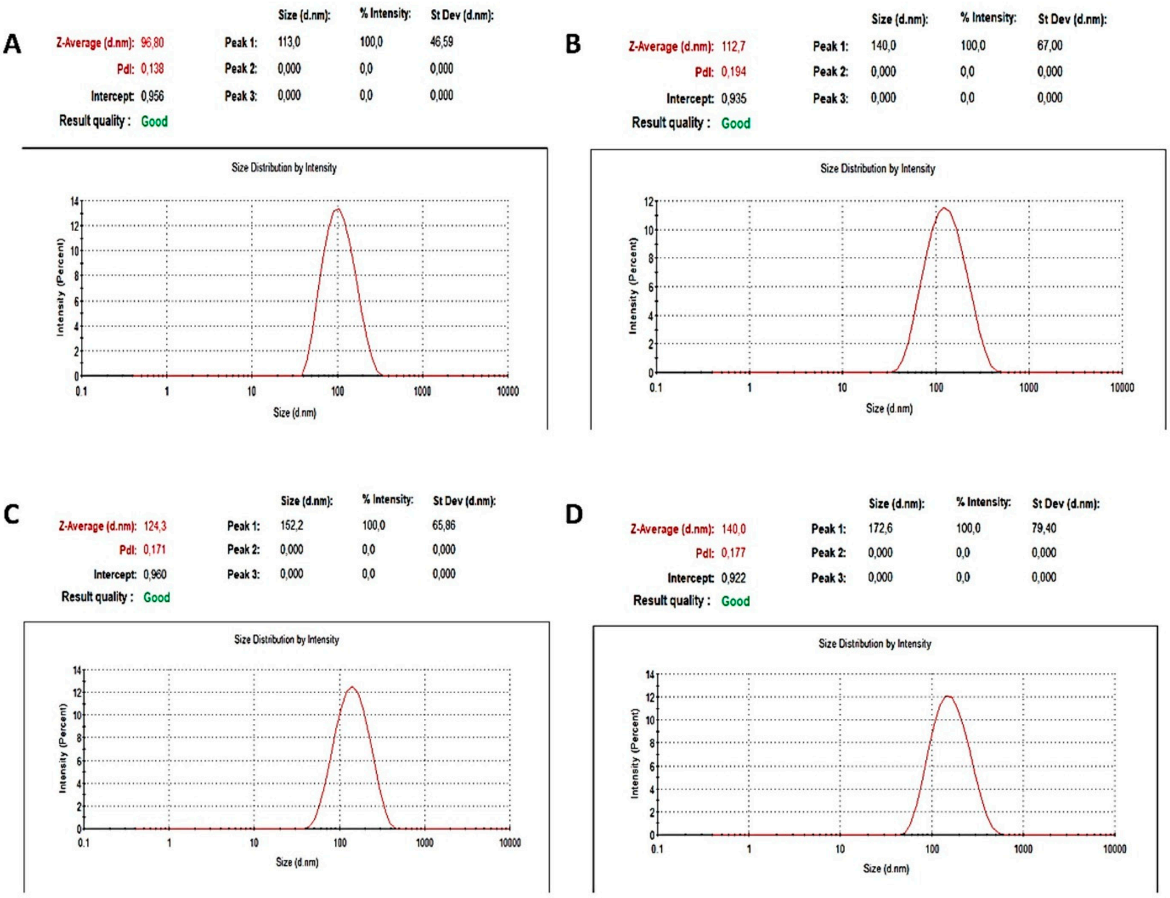

Figure S2. DLS size distribution curves of functionalized nanoclusters. (A) MNs2-PEG, (B) MNs3-PEG, (C) MNs2-PEG-OX26, (D) MNs3-PEG-OX26 demonstrating maintained narrow size distributions after surface functionalization. Curves are representative of triplicate measurements.

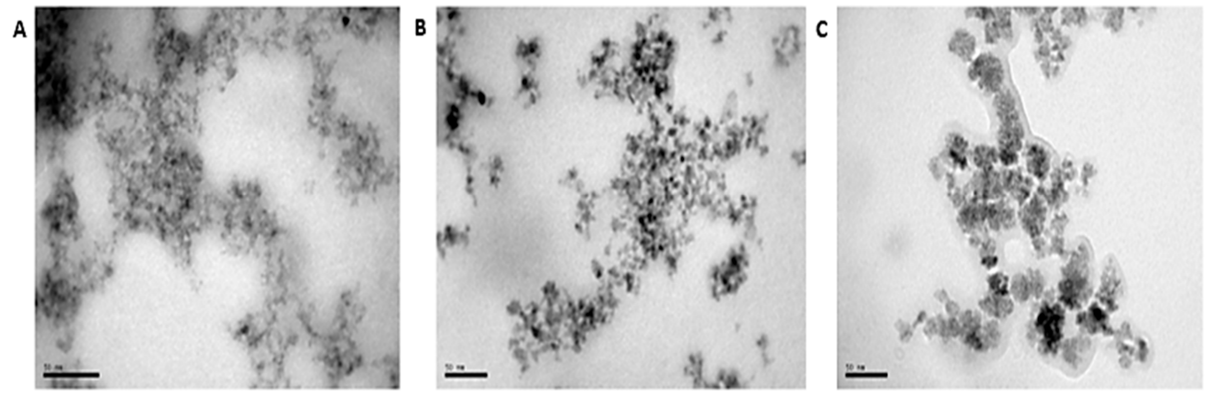

Figure S3. TEM morphological characterization of PAM-coated nanoclusters. (A) MNs1, (B) MNs2, and (C) MNs3 demonstrating systematic progression in cluster size and organization. Scale bars = 50 nm.

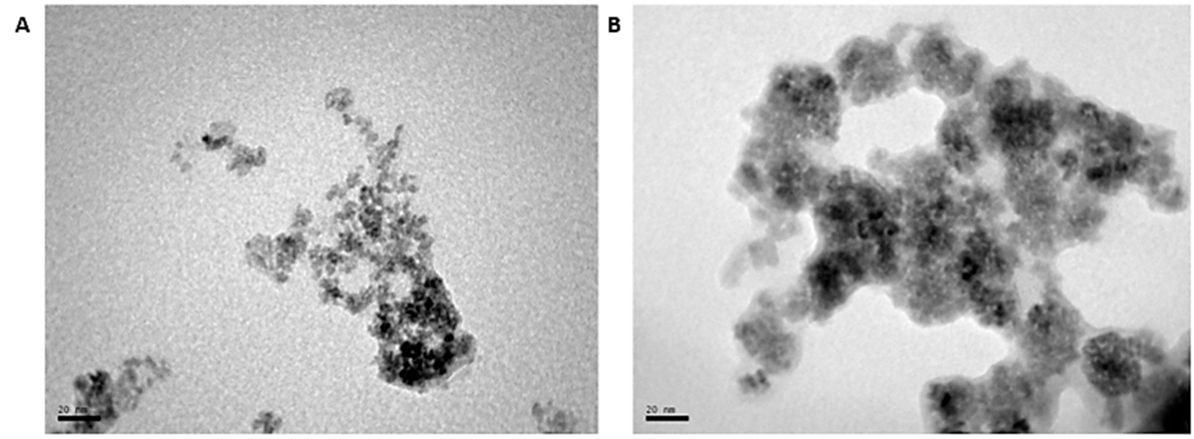

Figure S4. Higher magnification TEM analysis of nanocluster internal structure. (A) MNs2 and (B) MNs3 showing discrete primary SPION nanoparticles assembled within cluster frameworks, confirming multi-particle cluster formation. Scale bars = 20 nm.

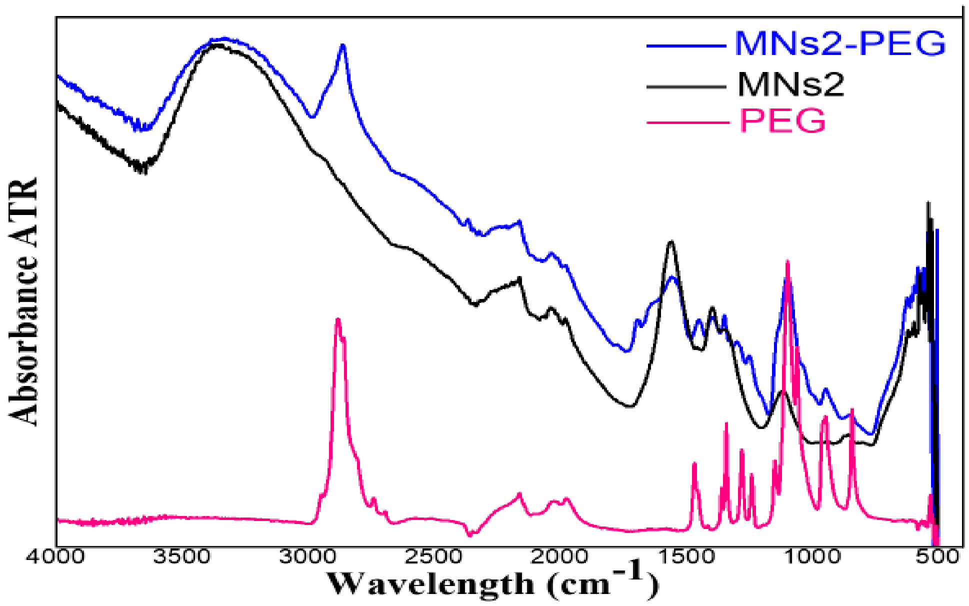

Figure S5. Characterization of MNs2-PEG. ATR FT-IR spectrum confirming the conjugation by the appearance of the PEG's C-H vibrations at ~2800-2900 cm^{-1} and and evidence of amide bond formation around 1698 cm^{-1}.

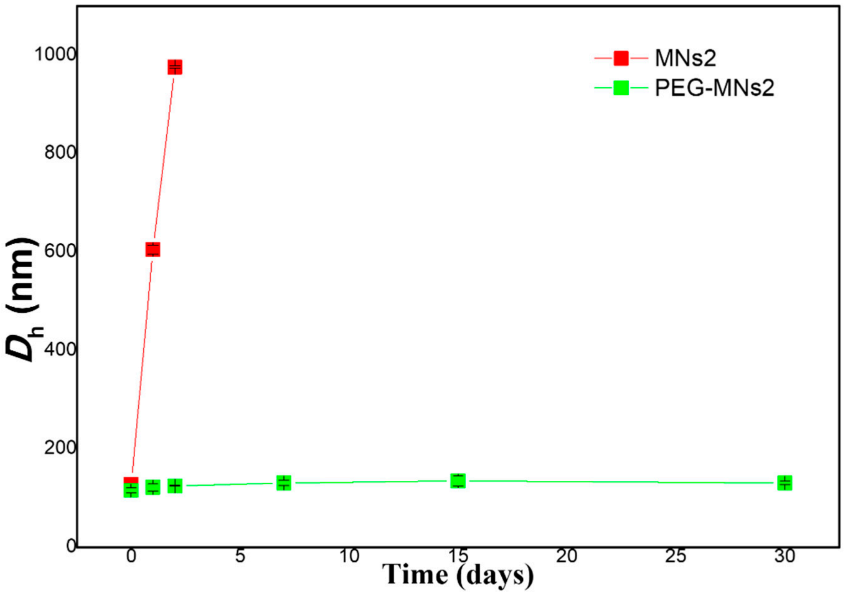

Figure S6. Stability study of MNs2-PEG *vs* non-PEGylated control (MNs2). Dynamic light scattering studies were performed for a period of 30 days in order to compare the long term colloidal behavior of MNs before and after PEGylation. PEGylated nano-crystallites remain stable even after 30 days, in contrast to the ones without PEG, which rapidly aggregate and precipitate

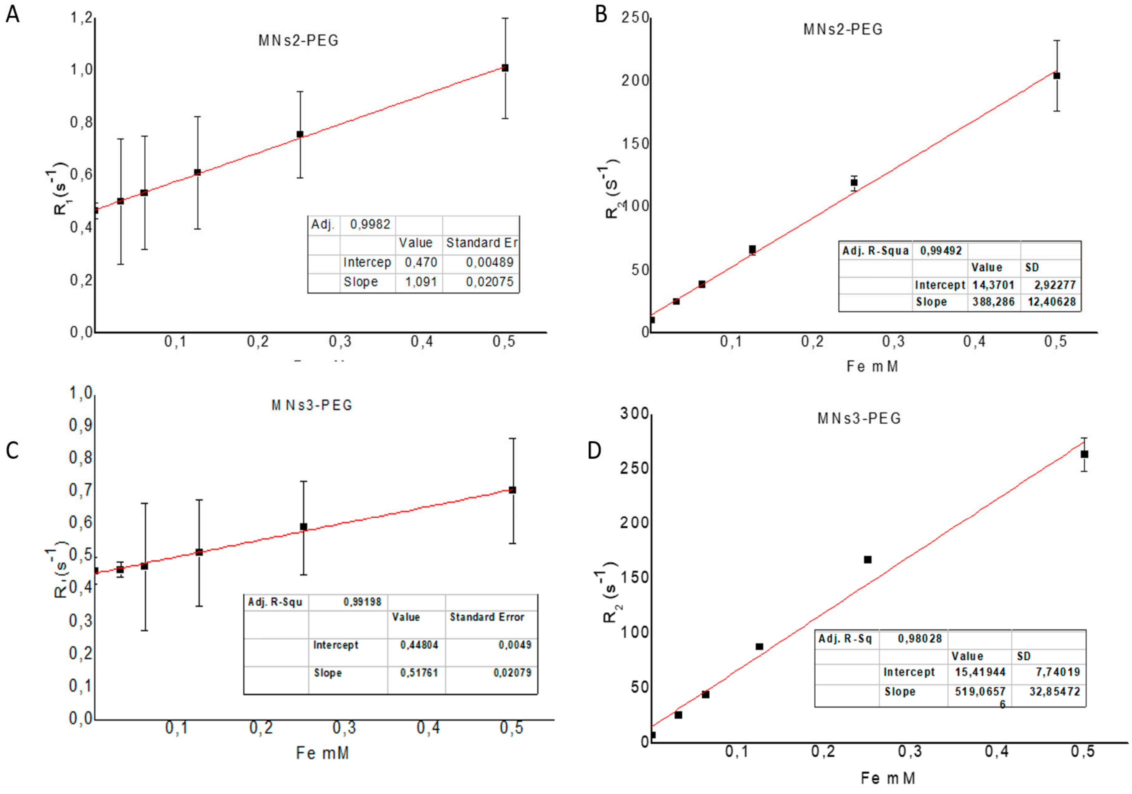

Figure S7. Linear regression analysis of longitudinal relaxivity (r_1) (**A, C**) and transverse relaxivity (r_2) (**B, D**) for MNs2-PEG and MNs3-PEG formulations measured at 7T in phantom agar. Error bars represent standard deviation from T_1/T_2 curve fitting. Regression parameters are shown in each panel.

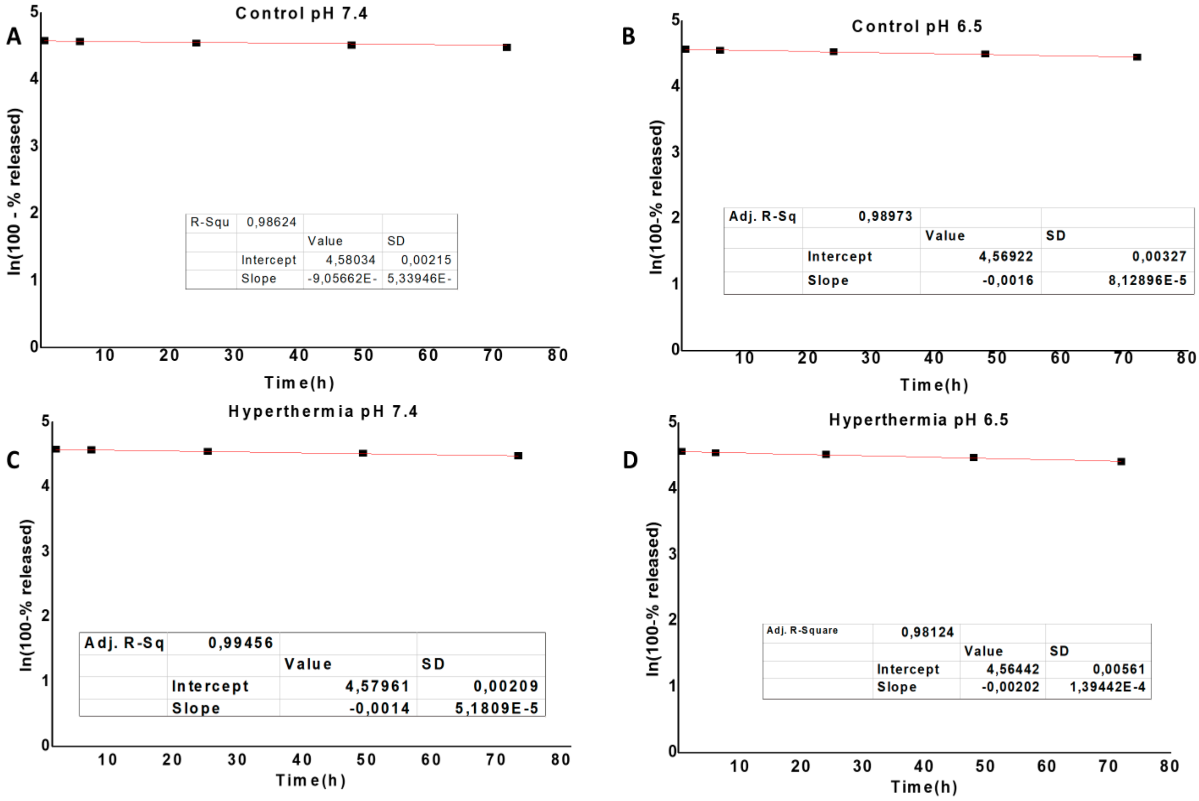

Figure S8. First order kinetics linearization plots for DOX release.Linear regression analysis showing ln(100 - % released) vs. time for (A) Control pH 7.4, (B) Control pH 6.5, (C) Hyperthermia pH 7.4, (D) Hyperthermia pH 6.5. High correlation coefficients ($R^2 > 0.98$) confirm first order release behavior across all conditions. Data points represent mean ± SD (n=3)

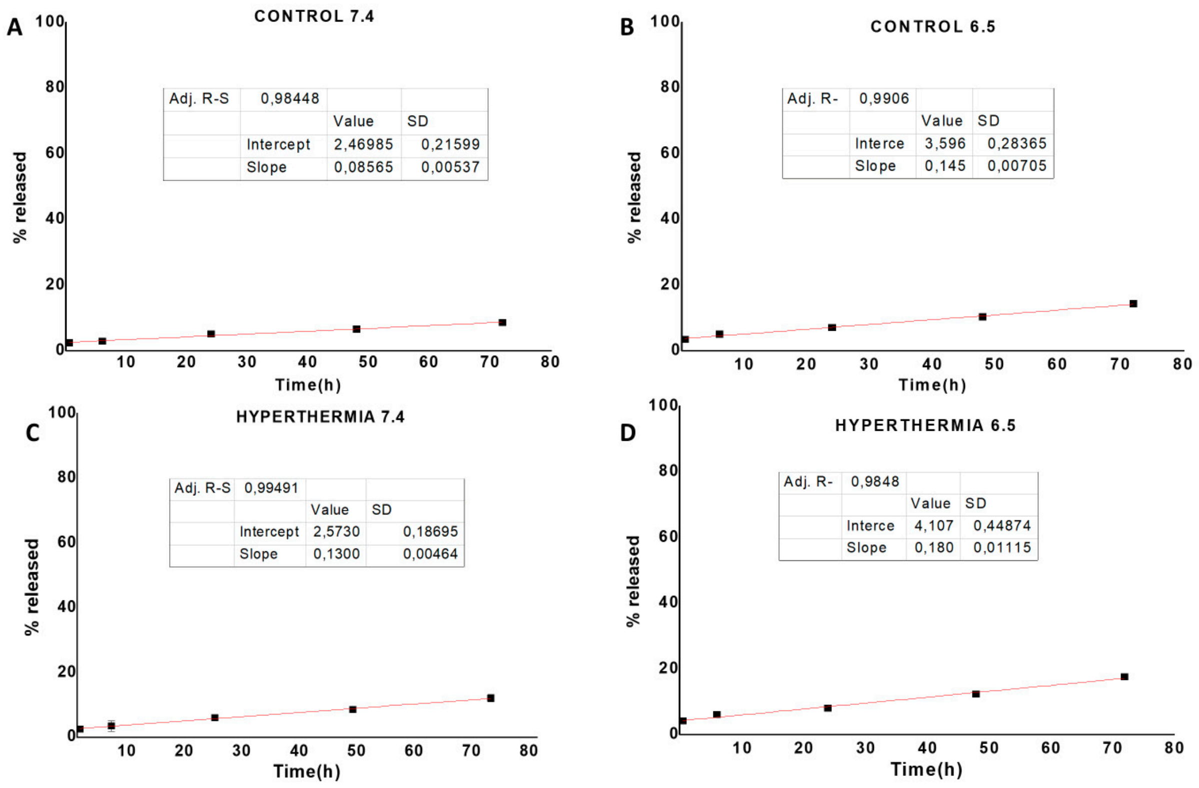

Figure S9. Zero order kinetics linearization plots for DOX release.Linear regression analysis showing % drug released vs. time for (A) Control pH 7.4, (B) Control pH 6.5, (C) Hyperthermia pH 7.4, (D) Hyperthermia pH 6.5. All conditions demonstrate excellent linear fit ($R^2 > 0.98$), indicating zero order release kinetics. Data points represent mean ± SD (n=3)
